# Case report: A gain-of-function of hamartin may lead to a distinct “inverse *TSC1*-hamartin” phenotype characterized by reduced cell growth

**DOI:** 10.3389/fped.2023.1101026

**Published:** 2023-03-30

**Authors:** Andrea D. Praticò, Raffaele Falsaperla, Mattia Comella, Giuseppe Belfiore, Agata Polizzi, Martino Ruggieri

**Affiliations:** ^1^ Unit of Clinical Paediatrics, Department of Clinical and Experimental Medicine, University of Catania, Catania, Italy; ^2^ Units of Neonatology and Neonatal Intensive Care and Paediatrics and Paediatric Emergency, Azienda Ospedaliero Universitaria “Policlinico”, Catania, Italy; ^3^ Unit of Paediatric Radiology, Department of Radiodiagnostics, Azienda Ospedaliero Universitaria “Policlinico”, Catania, Italy; ^4^ Chair of Paediatrics, Department of Educational Sciences, University of Catania, Catania, Italy

**Keywords:** TSC1, hamartin, tuberous sclerosis complex, microcephaly, brain cortical malformations

## Abstract

Mutations of *TSC1* and *TSC2* genes cause classical Tuberous Sclerosis Complex (TSC), a neurocutaneous disorder characterized by a tendency to develop hamartias, hamartomas, and other tumors. We herein report on a girl, now aged 5 years, who presented a previously unreported, distinct clinical phenotype consisting of primary microcephaly (head circumference = 40 cm, −5.6 standard deviations), brain anomalies including hypoplasia of the corpus callosum (with a residual draft of the genu), simplified parieto-temporal gyral pattern, colpocephaly with ectasia of the temporal ventricular horns, intellectual disability, and a general pattern of reduced growth (with weight and height < 3rd centiles). No classical features of TSC were recorded; the girl harbored a novel missense variant in *TSC1* (c.611G > A). We hypothesize that her clinical phenotype could be related to a “gain-of-function” of the *TSC1* protein product hamartin, causing an increase in the effects of the protein on inhibition of its intracellular targets (i.e., mTORC or RAC1 pathways), resulting in a distinct “inverse *TSC1*-hamartin” phenotype characterized by reduced growth of cells instead of the more classical predisposition to increased cell growth.

## Introduction

Mutations in *TSC1* and *TSC2* genes lead to abnormal production of hamartin and tuberin, two powerful suppressors of the mTOR pathway of cellular growth, causing the well-known Tuberous Sclerosis Complex (TSC) phenotype [MIM # 191100 and # 613254] (1–3). TSC is characterized by multi-organ involvement including multiple hypomelanotic macules, facial (e.g., forehead plaque), truncal (e.g., shagreen patches) and ungual fibromas, facial angiofibromas, dental enamel pits, multiple cardiac rhabdomyomas, angiomyolipomas and cysts of the kidney and lungs (and other organs), bone dysplasia and overgrowth, brain cortical tubers, subependymal nodules, linear white matter anomalies and subependymal giant cell astrocytoma, epilepsy, psychomotor delay/intellectual disability, and behavioral abnormalities with increased risk of manifesting autism spectrum disorders. Clinically, the range of involvement and phenotype severity is wide, even within families: affected persons may present only a few skin manifestations with null to minimal neurological involvement or a severe multi-organ and neurodevelopmental disorder with psychomotor delay, drug-resistant seizures, autism, and a predisposition to develop brain and other organ tumors.

The two proteins involved in the pathogenesis of TSC (i.e., *TSC1*/hamartin and *TSC2*/tuberin) cause a single disease, as their action is targeted, in “cooperation”, towards the same cellular pathway, primarily the mechanistic target of rapamycin complex 1 (mTORC1), and other complex inhibitory intracellular mediators or pathways of cellular growth and migration (e.g., RAC1 and RHO G-protein pathways). A direct genotype-phenotype correlation has been established in many studies, but sometimes a single gene variant has been linked to different phenotypes, thus suggesting that other genes or environmental/intracellular factors can contribute to the overall clinical phenotype (4–6). All the mutations and gene variants causing TSC have been shown to be “loss-of-function” mutations, causing impaired, reduced, or absent activity of one of the two proteins. Haploinsufficiency is another important aspect to consider, in that both *TSC1* and *TSC2* are expressed in an autosomal dominant fashion and a single mutation of a single copy is sufficient to cause the clinical phenotype.

To the best of our knowledge, no “hyperactivating” [i.e., gain-of-function] mutations of *TSC1* or *TSC2* have been reported to date in individuals with TSC; other diseases caused by mutation of genes involved in the synthesis of downstream effectors of *TSC1* and *TSC2* have been reported in recent years, including a neurocutaneous phenotype caused by RHOA inactivation and other clinical entities due to Rac1 inactivation, which present with under- or over-growth of the brain depending on the residual function of the protein involved (7–10).

We report on a girl presenting a disease-causing mutation of *TSC1* without stigmata of TSC, but with great similarity to the Rac1-related syndrome (i.e., a complex brain malformation syndrome, including severe primary microcephaly, simplified gyration pattern and other corpus callosum, and ventricle abnormalities). To explain, pathogenically, this phenotype, we hereby hypothesize a gain-of-function effect of the *TSC1* gene variant on hamartin and increased, rather than decreased, inhibition of the mTOR and/or Rac1 pathways, thus leading to the definition of a distinct (likely novel) phenotype with reduced brain cellular growth.

## Case report

The proband, a girl, is the only child of healthy, non-consanguineous parents. She was first referred to our Unit of Clinical Pediatrics at the age of 18 months for a diagnostic workup regarding her microcephaly. She was born at 39 weeks of gestation by caesarean section; the pregnancy was characterized by an ultrasonography diagnosis of microcephaly, reduced gyral pattern, and agenesis of the corpus callosum at gestational age 25 weeks. Serological screening for congenital infections (i.e., Cytomegalovirus, *Toxoplasma gondii*, Varicella-Zoster Virus, and Hepatitis C Virus) during pregnancy and at birth (in the mother and her daughter) was negative, as was the proband neonatal extensive screening for inborn errors of metabolism. Her birth weight was 2.6 kg, height was 47 cm, and head circumference was 32 cm (all below the 3rd percentile). Neonatal history was negative, and she was not breast-fed by the mother.

At our first diagnostic workup, at age 18 months, her weight was 8.4 kg (<3rd percentile), height was 74 cm (<3rd percentile), and head circumference was 38.5 cm (<3rd percentile, −5.6 standard deviations). Three small hyperpigmented maculae (1-cm diameter each) were present on her abdomen. The mother reported irritability and sleep disorders (insomnia). She underwent a new heart examination by means of ECG and echocardiography, abdominal ultrasound, and full ophthalmological examination, all of which were normal. Her eye-to-eye and social interaction with parents and other people was within normal limits, but she was not able to walk alone and spoke only a few words. Brain MRI and additional laboratory and instrumental investigations were planned but the family was temporarily lost to follow-up due to the Covid-19 pandemic.

**Figure 1:**
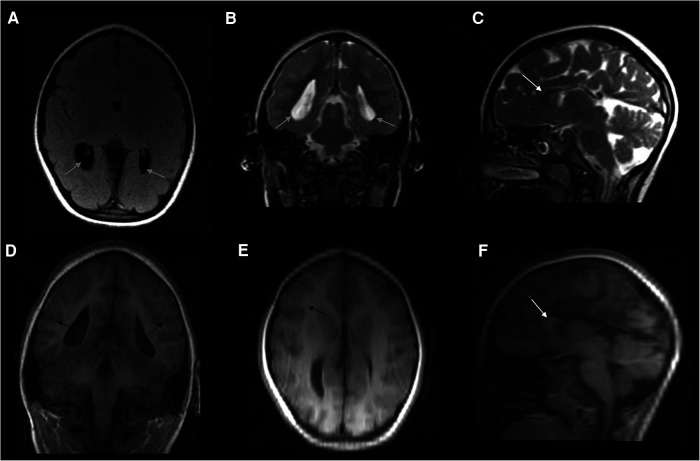
Composite panel of six neuroimages showing the brain MRI study in the girl, at the age of 3.5 years: FLAIR-axial scan (**A**), T2-weighted coronal (**B**), and sagittal (**C**) sections; and coronal T1 3D scan (**D**), as well as reconstructions (reformat) derived from this last study both in axial (**E**) and sagittal (**F**) planes. A complex picture of microcephaly, partial agenesis of the corpus callosum with remnants of the genu (white arrow), simplified gyral pattern especially in the parietal and occipital regions of both hemispheres; enlarged and flattened ventricles (grey arrows), with colpocephaly and ectasia of temporal horns were also present. Apparently, no changes in myelin composition and cortical thickness were noticed (black arrows), even if the image protocol shown in this figure is of low quality due to partial panel reformatting. No signs or stigmata of TSC were detected within the brain (e.g., cortical tubers).

At the age of 3.5 years, the family again referred the child for completion of her diagnostic work-up. At that stage, brain MRI showed microcephaly with a small brain, simplified parieto-temporal gyral pattern, hypoplasia of the corpus callosum, with a residual draft of the genu, and enlargement and flattening of the ventricles, with colpocephaly and ectasia of the temporal horns (Figure 1). At the age of 4 years, her general conditions were good. Her weight and height were 12.1 kg and 89 cm, respectively (<3rd percentile), head circumference was 40 cm (<3rd percentile, −5.6 standard deviations), the girl was able to walk, did not present any seizures, and showed moderate intellectual disability [IQ by means of WPPSI-III = 67; verbal comprehension index = 61; visual spatial index = 72; fluid reasoning index = 63; working memory index = 70; processing speed index = 69]. She was assisted by a support teacher at school and was under speech-language therapy twice a week.

The girl and her parents underwent next-generation sequencing (NGS) testing with a panel of 161 genes related to brain malformations. A novel variant (**c.611G > A**), in one copy of the *TSC1* gene, was found in the child, and not in her parents (interpreted as a *de novo* variant). An in-silico analysis was performed using **VarCards**, **ProAffiMuSeq,** and **Mutation Taster** tools (11–15) (see the following sections). Over the following months, the variant was confirmed by Sanger sequencing of the gene; the girl and her parents were also submitted to a further whole exome sequencing analysis, which confirmed the results of the NGS panel and failed to find additional variants in other genes. The *TSC1* variant was hypothesized to cause a gain-of-function effect on hamartin, and consequent hyperactivation of its downstream effectors.

## Genetic analysis

The gene panel for brain malformations consisted of 161 genes sequenced by NextSeq 500 System Sequencing [Table 1]. Sequencing was preceded by selective enrichment of the regions of the DNA analyzed, through hybridization with selectively designed probes (Sureselect, Agilent). The average coverage of the target bases was <100X.

**Table 1:** A list of the 161 genes included in the NGS panel for brain malformations searched in our patient.

Gene	Gene	Gene	Gene
*ACTB*	*CSPP1*	*MCPH1*	*SEPSECS*
*ACTG1*	*CUL4B*	*MFSD2A*	*SHH*
*ADGRG1*	*DAG1*	*MKS1*	*SIX3*
*AHI1*	*DCHS1*	*MTOR*	*SLC25A19*
*AKT1*	*DCX*	*MYCN*	*STAMBP*
*AKT3*	*DEPDCS*	*NDE1*	*STIL*
*AMPD2*	*DYNC1H1*	*NEDD4L*	*STRADA*
*ANKLE2*	*EML1*	*NFIX*	*TBC1D20*
*ARFGEF2*	*EMX2*	*NPHP1*	*TBC1D7*
*ARL13B*	*ERMARD*	*NPRL2*	*TBCD*
*ARX*	*EXOSC3*	*NPRL3*	*TCTN1*
*ASNS*	*EXOSC8*	*NSD1*	*TCTN2*
*ASPM*	*EZH2*	*OCLN*	*TCTN3*
*ATRX*	*FAT4*	*OFD1*	*TGIF1*
*B3GALNTZ*	*FIG4*	*OPHN1*	*TMEM138*
*B4GAT1*	*FKRP*	*ORC1*	*TMEM216*
*B9D1*	*EKTN*	*PAFAH1B1*	*TMEM231*
*C5orf42*	*FLNA*	*PCLO*	*TMEM237*
*CASK*	*FOXC1*	*PDE6D*	*TMEMS*
*CC2D1A*	*GLI2*	*PHC1*	*TMEM67*
*CC2D2A*	*GMPPB*	*PI4KA*	*TMTC3*
*CCND2*	*GNAQ*	*PIK3CA*	*TSC1*
*CDK5*	*GPSM2*	*PIK3R2*	*TSC2*
*CDK5RAP2*	*IER3IP1*	*PLK4*	*TSEN15*
*CDK6*	*INPPSE*	*POMGNT1*	*TSEN2*
*CDON*	*ISPD*	*POMGNT2*	*TSEN34*
*CENPE*	*KANSL1*	*POMT1*	*TSEN54*
*CENPJ*	*KAT6A*	*POMT2*	*TUBA1A*
*CEP135*	*KATNB1*	*PPP1R15B*	*TUBA8*
*CEP152*	*KIAA0556*	*PQBP1*	*TUBB*
*CEP290*	*KIAA0586*	*PTCH1*	*TUBB2A*
*CEP41*	*KIF11*	*PTEN*	*TUBB2B*
*CEP63*	*KIF2A*	*RAB18*	*TUBB3*
*CHMP1A*	*KIF5C*	*RAB3GAP1*	*TUBG1*
*CIT*	*KIF7*	*RAB3GAP2*	*VLDLR*
*CLP1*	*KNL1*	*RARS2*	*VPS53*
*CNTNAP2*	*LAMA2*	*RELN*	*VRK1*
*COL4A1*	*LAMB1*	*RPGRIP1L*	*WDR62*
*COL4A2*	*LAMC3*	*RTTN*	*YWHAE*
*CRADD*	*LARGE1*	*SASS6*	*ZIC1*
* *	* *	* *	*ZNF423*

The resulting variants were therefore analyzed using the BWA software (V.0.7.7-r441), Picard (v 1.109), and GATK (v 3.1), and annotation was performed using Annovar (v. 17June15). All the non-synonymous exonic variants and splice sites (±2 nucleotides of the coding exons) with frequencies lower than 0.1% for dominant genes and lower than 1% for recessive genes (ExAC, GnomAD) were considered in the analysis. Data obtained through NGS sequencing were therefore analyzed with the tool CoNVaDING in order to identify variants of copy number of the exons (14) and variants reported within the Human Genetic Mutation Database (HGMD) nomenclature. The novel variant (**c.611G > A**) found in one copy of the *TSC1* gene was found in the proband and not in her parents.

## In-silico analysis of the *TSC1* gene variant

The *TSC1* variant **c.611G > A** recorded herein is associated with replacement of an arginine residue with histidine (both amino acids harbor “basic” side chains) in position 204 of the protein hamartin (R204H). The change occurs in exon 8, within the “rho-activating domain” (aa 145–510) of hamartin, close to the tuberin-binding site (aa 302–430). No other variants were found in the remaining genes.

This R204H amino acid substitution was analyzed using different in silico-tools.

Its effects on *hamartin function* were predicted as deleterious by 22/23 tools in the **VarCards** prediction software [which analyses the effects of a gene variant in a set of 23 tools], with an overall damage score of 0.96 [range 0–1].

Such results, however, do not provide accurate information on potential *gain, loss*, *or changes of function*, thus the “mutated” protein was further tested using the **ProAffiMuSeq** tool (11, 13), a webserver that calculates the binding free energy change (*ΔΔ*G in kcal/mol) for mutant protein-to-protein complexes. Using this tool, we analyzed the *affinity* of the “mutated” hamartin to tuberin (enzyme complex with non-inhibitor models) and to Rho-GTPase Rac1 (protein-inhibitor model) by comparing the recorded variant and substitution at position 204 to two known variants and substitutions occurring at position 204, which are related to classical TSC phenotypes (R204C and R204P). In all these three genetic “models” (R204P, R204C, and R204H) the affinity of hamartin to Rac1 was decreased, with substantial differences in terms of ΔΔG (4.49, 2.03, and 0.54, respectively). The interaction with tuberin gave contrasting results: R204P and R204H showed increased affinity (ΔΔG was −0.21 and −0.04, respectively), while R204C showed decreased affinity (ΔΔG 1.39) [Table 2].

**Table 2:** In silico prediction of the effects of protein changes at position 204 of hamartin. The first model evaluates the affinity of the mutated hamartin (inhibitor) to RAC1 (protein); the second reports on the effects on the interactions of hamartin and tuberin; the third model is taken from *mutationtaster* and evaluates the effects of the substitution of the new amino acid in comparison to the original protein (score 0–150) [see text for explanation].

			Effects on Hamartin-Rac1 Interaction		Effects on Hamartin-Tuberin Interaction		Effect on Hamartin Function
			Protein-inhibitor model		Enzyme complex with non-inhibitor		Amino acid score (Mutation taster)
Protein change	Gene mutation	Phenotype	Predicted ΔΔG	Affinity	Predicted ΔΔG	Affinity	
R204P	c.611G > C	TSC	4.49	Decreased	−0.21	Slightly increased	103
R204C	c.610C > T	TSC1/FCD	2.03	Decreased	1.39	Decreased	180
**R204H**	**c.611G > A**	**Present patient**	**0.54**	**Slightly decreased**	**−0.04**	**Slightly increased**	**29**

TSC, Tuberous sclerosis complex; FCD, Focal cortical dysplasia. ΔΔG, binding free energy change (Kcal/mol).

The three tested genetic variants/substitutions (R204P, R204C, and R204H) were further analyzed using **Mutation Taster**, which compares the predicted “*amino acid change” score*: this latter change is taken from an amino acid substitution matrix (Grantham Matrix) (12), which takes into account the physical-chemical features of amino acids and scores substitutions according to the degree of difference between the original and the new amino acid. Scores may range from 0.0 to 215. The variant/substitution detected herein (i.e., R204H) had a score of 29 (mild effect on hamartin function), while the two other mutations had scores that were 3 to 6 times higher [Table 2].

## Discussion

**Figure 2:**
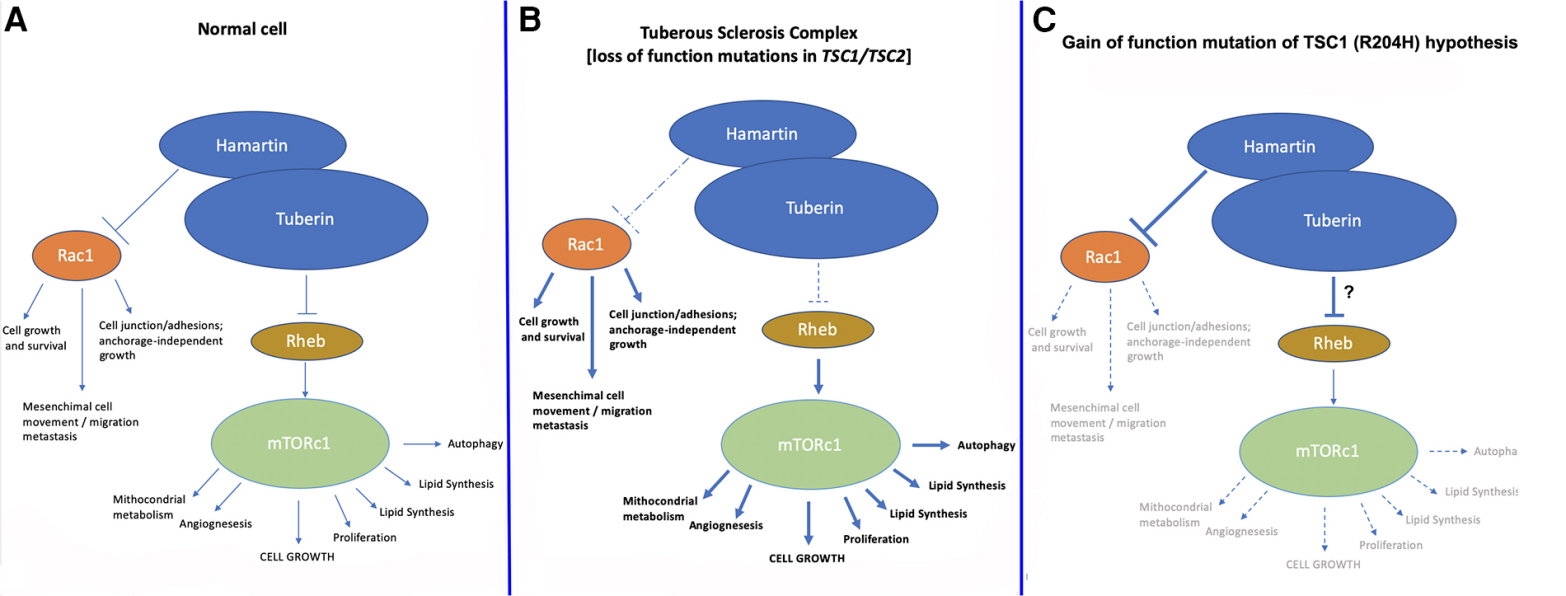
Normal pathway of hamartin/tuberin inhibition of mTORC1 and Rac1 pathways (**A**) compared to (**B**) the current TSC model and (**C**) the “gain-of-function of hamartin” hypothesis postulated in the case described herein. We hypothesize that in the present case, increased inhibition of mTORC1 and/or Rac1 led to the phenotype of reduced cerebral growth; another explanation could be found in the increased interaction between hamartin and tuberin leading to increased inhibition of mTORC1. In both cases, a substantial reduction in the growth and replication of neuronal cells in a very early phase during embryonic/fetal life likely led to the clinical manifestations recorded in our patient, which in turn may represent an “*inverse/reverse TSC1* phenotype”.

*TSC1* and *TSC2 loss-of-function* mutations/variants lead to impaired function of the hamartin/tuberin complex (which is a dimer) and in turn to the classical TSC phenotype. The increased oncological risk of TSC has been explained by the reduced inhibition of the mTOR pathway, which is a very strong activator of protein synthesis and cell growth (1–6). Hamartin and tuberin inhibit cell growth through the activity of a complex system of GAP (GTPase-activating protein), specifically the Rheb (Ras homologue enriched in brain) protein, which is dephosphorylated into its GDP-form, and which ultimately inhibits mTORC1 (mammalian/mechanistic target of rapamycin complex 1) (Figures 2A,B). In physiological conditions, mTORC1 activation promotes cell growth, at least in part by increasing the anabolic process of protein synthesis through activation of S6K and inhibition of 4E-BP (3).

Other effects may be due to reduced inhibition of the RHOA and Rac1 pathways, which are essential in cell growth and survival, mesenchymal cell movements, and migration and metastasis of cells, as well as in cell junctions and adhesion maintenance. Additional effects on angiogenesis and cell barrier integrity have also been reported (7–9, 15–17).

In our patient, who harbored a novel *TSC1* variant, the phenotype was not compatible with classical TSC, even considering the wide variability of the clinical spectrum of the disease (1). VarCards aggregator of *in-silico* models showed that the mutation/variant recorded herein and occurring within domain 204 of hamartin was a disease-causing one. The site [amino acid 204, exon 8] was within the binding site of tuberin to Rho proteins; this domain extends from amino acid 145 to 510 and contains the tuberin-binding site [aa. 302–430]. The closest upstream phosphorylation site is at position 357 and is regulated by kinase GSK3*β*.

Given the fact that in-silico prediction models “roughly” analyze the protein sequence and report if a missense mutation may give rise to a different protein, but are “unable” to properly analyze a possible “gain-of-function”, we compared the mutation/amino acid substitution R204H to two other “known” variants/substitutions (i.e., R204P and R204C), occurring within the same amino acid and causing classical TSC, to the binding properties of the “mutated” hamartin with Rac1 and tuberin proteins. Comparison and analysis of these three models provided intriguing results in that all three models showed decreased hamartin affinity to Rac1. Notably, this decrease in affinity, in terms of ΔΔG, was very strong for R204P and R204C but milder for R204H (0.54); the affinity with tuberin (the binding domain of which is very close to the mutated region recorded herein, at position 302–430) showed different results, with some increase in affinity for R204P and R204H (-0.21 and −0.04, respectively) and a decrease for R204C (1.39). The “milder” changes in R204H affinity could be attributed to the “properties” of the two “substituted” amino acids (the original arginine with histidine), which both have basic lateral chains and similar biochemical properties, including a comparable hydrophobic index (−3.2 His, −4.5 Arg), and similar molecular weights (155.1 Da His, 174.2 Da Arg).

The (mildly) decreased affinity to Rac1 could be balanced by the increased binding properties of hamartin to tuberin, which would therefore strengthen the action of the complex “against” the mTOR activation cascade. As a consequence, the variant hereby recorded would not have the “classical” reduction in function seen in the TSC1/TSC2 dimer complex, although it could have increased the effects on mTOR inhibition and secondarily the Rac1 pathway.

To further confirm the mild effects in terms of loss-of-function of the selected protein, the amino acid score calculated by Mutation Taster gave a “mild” result of 29 (range 0–215), compared to the higher results of the two other mutations/amino acid substitutions (i.e., R204P 103 and R204C 180). In this setting, R204H could be interpreted as a “non-severe” variant that could have some gain-of-function effects on the hamartin-tuberin complex, thus justifying the clinical phenotype seen in our patient, which comprised primary microcephaly (a malformation with reduced cellular growth/increased apoptosis), simplified gyration, and stunted overall growth (weight and height). In this patient, microcephaly can be regarded as “primary” given its prenatal recording and its manifestations at birth and during follow-up, as well as the association with a pattern of simplified gyration and sulci, in particular in the parietal and occipital regions (Figure 1). Microcephaly does not seem to be acquired, as the mother did not report the use of any drug or substances causing microcephaly *in utero*, nor was there evidence of any congenital infection during pregnancy (18).

The effects of increased inhibition on the mTOR pathway, including microcephaly, have been reported in animal models. In mice, Zhang and colleagues (19) found that inactivation of mTORC1 signaling in postnatal neurons induced reactive astrocyte gliosis. They studied knock-out mice for mTORC1 complex and found that inactivation of mTORC1 in neuronal progenitors impaired the growth and proliferation of neurons and astrocytes, resulting in a smaller brain and death shortly after birth. In another animal model (20), a transgenic mouse expressing a gain-of-function mutant of mTOR in the forebrain selectively hyperactivated mTORC1 during the embryonic stages with subsequent cortical atrophy, causing prominent apoptosis of neuronal progenitors, associated with upregulation of HIF-1*α*; curiously, and unlike the study by Zhang and colleagues, in postnatal and adult stages, the same hyperactivation resulted in a TSC-similar phenotype with cortical hypertrophy and severe seizures.

In humans, two different phenotypes of brain-reduced growth have been linked to RhoA and Rac1 GTPase, which are critically modulated by hamartin and tuberin (21). These proteins are members of the Ras superfamily of low molecular weight, monomeric G- proteins: in particular the RhoA, Rac1, and Cdc42 proteins are key regulators of actin cytoskeletal dynamics, and modulate other important cell functions including membrane trafficking, cell cycle progression, gene transcription, adhesion, migration, and survival. A new mosaic neuroectodermal dysplasia syndrome caused by inactivation of the RHOA pathway (which is strictly linked to hamartin inhibition) has been reported [**EDFAOB**, MIM # 618727], presenting with **e**ctodermal **d**ysplasia in the form of linear hypopigmentation along the lines of Blaschko and alopecia, **f**acial dysmorphisms (e.g., microstomia, malar hypoplasia, down-slanting palpebral fissures and broad nasal bridge), **a**cral (e.g., brachydactyly, syndactyly, broad great toe), **o**cular (e.g., microphthalmia, myopia, strabismus), dental (e.g., oligodontia, microdontia, conical teeth, abnormal enamel), and **b**rain anomalies including apparently asymptomatic diffuse cortical leukoencephalopathy with ventricular enlargement (10, 15). Individuals with mutations occurring in genes involved in the Rac1 pathway (*RAC1, TRIO)* (7–9, 18) have been found to show distinct phenotypes, which include intellectual disability and significant variation of head circumference size; in particular, hypo-activating mutations/variants of Rac1 have been linked to severe microcephaly, while constitutively active substitutions caused macrocephaly. These findings demonstrate that Rac1 is strongly linked to cerebral size (9). Interestingly, mutations in the *TRIO* gene (whose protein product modulates the Rac1 pathway) have similar effects on brain size: macrocephaly or microcephaly depending on the residual functionality of the protein (7, 8).

We hypothesize that the herein-presented *TSC1* variant may have acted as a gain-of-function model of inhibition of the Rho-GTPase-Rac1 pathway (Figure 2C), leading to a somewhat “inverse *TSC1*-hamartin” phenotype. Other explanations could be increased interaction between hamartin and tuberin leading to increased inhibition of mTORC1 with the substantial reduction of growth and replication of neuronal cells in a very early phase during embryonic/fetal life.

The present study has some limitations: a more powerful tool to prove or disprove pathogenicity would involve the creation of mouse/rat models harboring the same variant and recapitulating the phenotype; on the other hand, in-silico models like those used for the present study are becoming more practical, easy, and rapid ways to predict the function of a mutated protein.

Moreover, the quality of the imaging protocol (in particular and coronal T1 3D scan and its reformatted images, Figures 1E,F) is low and no longitudinal imaging evaluation nor advanced imaging techniques that better evaluate the CC have been performed so far in the patient.

## Data Availability

The datasets presented in this article are not readily available because of ethical and privacy restrictions. Requests to access the datasets should be directed to the corresponding authors.
